# New Formulation of Paraquat: A Step Forward but in the Wrong Direction?

**DOI:** 10.1371/journal.pmed.0050058

**Published:** 2008-02-26

**Authors:** D. Nicholas Bateman

## Abstract

The author discusses whether the new paraquat formulation introduced in Sri Lanka is a step forward in reducing deaths from paraquat self-poisoning.

Paraquat is a contact herbicide (one that kills plants by contact rather than being taken up in the roots and acting systemically) that is extensively used in agriculture, as it is fast-acting and non-persistent in the environment. It has, however, one major problem—its toxicity when ingested. The mechanisms of this toxicity are well understood and were described in detail over 20 years ago [1]. Paraquat is marketed for agricultural use as a concentrated solution, and in this form it is estimated that as little as a mouthful ingested is likely to be fatal.

Blood levels of paraquat are indicative of outcome [2], but many patients have levels over 100 times the estimated lethal concentration, making treatment extremely difficult. In these patients, death may occur within a few hours from multi-organ toxicity. In less severe cases renal failure and gastrointestinal upset occur, resulting in death within two or three days if untreated. If these complications are managed by haemodialysis and fluid resuscitation, pulmonary fibrosis follows due to redox recycling (repetitive oxidation and reduction of the molecule, using up cellular energy) of paraquat in the lung. Death is then secondary to anoxia several days later; early oxygen treatment is thought to *increase* the risk of lung damage as it fuels the redox process.

Twenty years ago it was clear that treatments for paraquat poisoning were ineffective and that new approaches to managing such poisoning were required [3]. In the intervening period, small-scale studies have suggested a potential role for immunosuppressants [4] in preventing death from lung injury, but the efficacy of this treatment remains in doubt [5].

**Linked Research Article**

This Perspective discusses the following new study published in *PLoS Medicine:*

Wilks MF, Fernando R, Ariyananda PL, Eddleston M, Berry DJ, et al. (2008) Improvement in survival after paraquat ingestion following introduction of a new formulation in Sri Lanka. PLoS Med 5(2): e49. doi:10.1371/journal.pmed.0050049

Martin Wilks and colleagues compared the outcome of paraquat self-poisoning with the standard formulation against a new formulation following its introduction into Sri Lanka.

## Is the New Paraquat Formulation Safer?

The agribusiness company Syngenta has attempted to go back to a first principle of poisoning prevention: that is, to change the formulation of the marketed product in an attempt to reduce human systemic exposure, and hence toxicity. Although paraquat has long contained an emetic, Syngenta has increased its concentration, added a purgative (magnesium sulphate), and included an alginate designed to cause gel formation of ingested product and thus delay absorption, with the intention of giving the emetic more opportunity to work.

In this issue of *PLoS Medicine,* Martin Wilks (of Syngenta Crop Protection AG) and colleagues report the results of an impressive clinical study in Sri Lanka of the new formulation [6]. Data on exposure, treatment, and outcome of patients who ingested paraquat were prospectively collected at nine hospitals from December 2003 to January 2006. The new paraquat formulation was introduced in October 2004. The identity of the formulation ingested after October 2004 was determined by blood or urine samples. The researchers compared mortality in those admitted during a control period before October 2004 (i.e., those who ingested the old paraquat formulation) with mortality in those who ingested the new product. The primary outcome measure was survival to three months.

Of the 297 patients who ingested the standard formulation, there were 221 deaths, while of the 289 patients who ingested the new formulation, there were 186 deaths. The mortality rate at three months was 72.9% for those who ingested the standard formulation compared with 63.3% for those who ingested the new formulation. Median time to death changed from only 0.9 days to 1.5 days after introducing the new formulation. Such a rapid progression to death indicates the magnitude of the doses ingested in this patient group, both before and after formulation change. The net overall effect of the change was to produce one more survivor for every ten patients poisoned. This would appear impressive, if it were not for the fact that between six and seven of every ten patients who ingest the new formulation still die.

## A Step in the Wrong Direction?

Although the reasons for the multiple formulation changes were clear to the authors prior to study commencement, these reasons are not well presented in their paper, making this reader feel their logic was perhaps confused. A recent study of paraquat plasma kinetics in rabbits shows that inclusion of alginate causes a reduction in paraquat absorption in vivo in this species—the area under the curve (0–24 h) was reduced from 33.8 +/− 3 microg.mL^−1^h for paraquat (Gramoxone) to 12.5 +/− 6 microg.mL^−1^h with an alginate formulation [7]. To be effective clinically, this magnitude of effect would be most likely to work in patients near to a fatal threshold dose. In the present study, the median mortality time in the baseline period was less than one day, indicating a very large median ingested dose of concentrated paraquat. Most European toxicologists will have seen relatively few patients who die in this very short time frame, as most patients take lesser doses and die from the later pulmonary fibrosis.

There was some internal inconsistency in the approaches used to alter the formulation, in that gastric emptying was intended to be delayed by the use of an alginate that would gel in gastric acid. However, since emetics work predominantly after passive absorption from the small bowel, their absorption is necessarily coincident with the active uptake of the paraquat in the small bowel. Emetics may also alter small bowel motility by inducing reverse peristalsis—hence the overall approach seems a little confused. Uptake of paraquat from the small bowel is rapid, and it was perhaps optimistic to think that magnesium sulphate would prevent absorption of sufficient quantity of paraquat by speeding its passage through the small bowel in this Sri Lankan patient group.

Quantities of paraquat ingested in this study were very substantial, and the overall approach of this new formulation would only be effective if a very high proportion of patients are very close to the borderline between a lethal and non-lethal dose—much lower than the median doses ingested by patients in this study. Although the change in paraquat formulation might have had an impact in Europe, where lower doses are usually ingested, such a change is now not needed as legislation has limited general access. As happened in the United Kingdom, Europe will remove the product completely [8]. In the more affluent countries of the world, toxicity prevention by product ban may be achievable, but seems less readily acceptable in the economic margins of subsistence farming.

## Where to Go From Here: A Signpost for the Future?

Wilks and colleagues' study clearly shows the difficulty of a harm-reduction campaign based on formulation change for paraquat in this environment. Prevention strategies are not however an impossible objective. If paraquat use is to continue, and we are told it will in Asia for the foreseeable future, then the answer must be to make it more difficult for the product to be swallowed in the heat of an emotional crisis.

Storage away from the home is one target for prevention, but use of communal locked storage facilities and independent keys for access may not be accepted and hence may be ineffective [9]. A second target would be attempts to further address formulation and packaging. Using the alginate to gel the concentrate might be one approach, making rapid swallowing difficult. Mixing would be required prior to application.

A final, more easily deliverable target, given the evident toxicity of the high concentrates used in Sri Lanka, is to consider using more dilute paraquat preparations as the primary product for agricultural use. It appears that such an approach is now being implemented in Sri Lanka [10]. This approach has implications for transport, both to and within the farm, but such a price seems worth paying—if it would reduce the number of early untreatable patients reported here and perhaps prevent so many young and needless deaths. All new approaches need testing, and Wilks and colleagues have the expertise needed for such evaluation.

## The Take-Home Message

Paraquat remains a poison for which there is still little strong evidence of effective therapies. It has caused significant mortality worldwide for over 30 years. If it is to remain in use, then studies such as the one by Wilks and colleagues are important. In retrospect, it can easily be seen that the new paraquat formulation was unlikely to be a major advance in this environment. We should therefore use the findings of the new study to focus attention on simple strategies to reduce paraquat-induced death (and morbidity from this and other pesticides) in Asia. Immunosuppressants after overdose are not the solution.
